# Pharmacological preconditioning with inhaled nitric oxide (NO): Organ-specific differences in the lifetime of blood and tissue NO metabolites

**DOI:** 10.1016/j.niox.2018.08.006

**Published:** 2018-11-01

**Authors:** Yasuko Nagasaka, Bernadette O. Fernandez, Andrea U. Steinbicker, Ester Spagnolli, Rajeev Malhotra, Donald B. Bloch, Kenneth D. Bloch, Warren M. Zapol, Martin Feelisch

**Affiliations:** a Anesthesia, Critical Care and Pain Medicine, Massachusetts General Hospital, Harvard Medical School, Boston, MA, USA; b Division of Metabolic and Vascular Health, Warwick Medical School, University of Warwick, Coventry, UK; c Clinical & Experimental Sciences, Faculty of Medicine, University of Southampton, Southampton General Hospital, Southampton, UK; d Department of Anesthesiology, Intensive Care and Pain Medicine, University Hospital Münster, University of Münster, Münster, Germany; e Cardiology Division of the Department of Medicine, Massachusetts General Hospital, Harvard Medical School, Boston, MA, UK; f Division of Rheumatology, Allergy and Clinical Immunology, Department of Medicine, Massachusetts General Hospital, Harvard Medical School, Boston, MA, USA

**Keywords:** Nitric oxide, Ischemia-reperfusion injury, NO metabolites, Cardioprotection, NO inhalation, Cardiac surgery, AAR, area at risk, AAR/LV, area at risk as a fraction of left ventricle, CAD, coronary artery disease, cGMP, cyclic guanosine monophosphate, iNO, inhaled nitric oxide, I/R, ischemia-reperfusion, LV, left ventricle, MI, myocardial infarction, MI/AAR, myocardial infarction as a fraction of area at risk, NO, nitric oxide, NO-heme, nitrosylheme, NO_2_, nitrogen dioxide, NOx, sum of nitrite and nitrate, RI, reperfusion injury, RNNO, N-nitrosamine, RSNO, S-nitrosothiols

## Abstract

**Background:**

Endogenous nitric oxide (NO) may contribute to ischemic and anesthetic preconditioning while exogenous NO protects against ischemia-reperfusion (I/R) injury in the heart and other organs. Why those beneficial effects observed in animal models do not always translate into clinical effectiveness remains unclear. To mitigate reperfusion damage a source of NO is required. NO inhalation is known to increase tissue NO metabolites, but little information exists about the lifetime of these species. We therefore sought to investigate the fate of major NO metabolite classes following NO inhalation in mice *in vivo*.

**Methods:**

C57BL/6J mice were exposed to 80 ppm NO for 1 h. NO metabolites were measured in blood (plasma and erythrocytes) and tissues (heart, liver, lung, kidney and brain) immediately after NO exposure and up to 48 h thereafter. Concentrations of S-nitrosothiols, N-nitrosamines and NO-heme products as well as nitrite and nitrate were quantified by gas-phase chemiluminescence and ion chromatography. In separate experiments, mice breathed 80 ppm NO for 1 h prior to cardiac I/R injury (induced by coronary arterial ligation for 1 h, followed by recovery). After sacrifice, the size of the myocardial infarction (MI) and the area at risk (AAR) were measured.

**Results:**

After NO inhalation, elevated nitroso/nitrosyl levels returned to baseline over the next 24 h, with distinct multi-phasic decay profiles in each compartment. S/N-nitroso compounds and NO-hemoglobin in blood decreased exponentially, but remained above baseline for up to 30min, whereas nitrate was elevated for up to 3hrs after discontinuing NO breathing. Hepatic S/N-nitroso species concentrations remained steady for 30min before dropping exponentially. Nitrate only rose in blood, liver and kidney; nitrite tended to be lower in all organs immediately after NO inhalation but fluctuated considerably in concentration thereafter. NO inhalation before myocardial ischemia decreased the ratio of MI/AAR by 30% vs controls (p = 0.002); only cardiac S-nitrosothiols and NO-hemes were elevated at time of reperfusion onset.

**Conclusions:**

Metabolites in blood do not reflect NO metabolite status of any organ. Although NO is rapidly inactivated by hemoglobin-mediated oxidation in the circulation, long-lived tissue metabolites may account for the myocardial preconditioning effects of inhaled NO. NO inhalation may afford similar protection in other organs.

## Highlights

• Breathing 80 ppm NO for 1 h increases NO metabolite concentrations, except nitrite, in murine blood/tissues.
• After NO inhalation, elevated NO metabolites return to baseline in a compartment-specific manner.
• Cardiac S-nitrosothiol and NO-heme levels remain elevated for ∼1 h after discontinuation of NO treatment.
• Breathing 80 ppm NO for 1 h just before cardiac ischemia reduces reperfusion injury in mice.

## 1 Introduction

Coronary artery disease (CAD; also known as ‘ischemic heart disease’) is the most common type of cardiovascular disease, and most deaths from CAD are caused by a heart attack [1]. About 790,000 people in the US have a heart attack each year, and 114,000 of those die [2]. In the UK, nearly 200,000 annual hospital visits are due to heart attacks [3]. When patients present with an acute myocardial infarction (MI), timely reperfusion (by either surgical/percutaneous coronary intervention or thrombolysis) is the most effective treatment to limit infarct size, which preserves left-ventricular systolic function and either prevents development or delays the onset of heart failure. Although reperfusion is essential to salvage myocardial tissue, paradoxically restoring blood flow after a period of vascular occlusion and tissue ischemia itself causes harm, a process known as myocardial reperfusion injury (RI). Since there is currently no effective prevention against RI there is ongoing interest in the use of adjunctive cardioprotective agents and procedures to be applied before/during myocardial reperfusion to limit infarct size [[4], [5], [6]]. Such protective measures may be particularly valuable in the peri-operative setting for patients undergoing cardiopulmonary bypass for cardiac surgery.

Powerful endogenous processes protect the heart (and other organs) from ischemia/reperfusion (I/R) damage. The phenomenon of ischemic preconditioning by which repeated, brief coronary occlusions preceding a longer occlusion markedly reduces MI size, was first described in dogs by Murray et al., in 1986 [7]; it has since been demonstrated in all species including humans. Despite the enormous research interest this landmark observation triggered, the precise molecular mechanisms and mediators involved remain incompletely understood. However, one of them appears to be nitric oxide (NO) [4,5], which has also been invoked in anesthetic preconditioning [8]. Consistent with this notion, myocardial RI is exacerbated in the absence of endothelial nitric oxide synthase [9], whereas cardiomyocyte-specific eNOS overexpression limits RI [10]. Accordingly, NO-donors and NO itself have been used as pharmacological preconditioning mimetics and demonstrated to be cardioprotective in animal experimental models [[11], [12], [13], [14]]. The current challenge is to translate this treatment paradigm from the laboratory bench to the bedside [15,16], with variable success so far in clinical studies. The reasons for the apparent failures are not always obvious but may include ineffective bioactivation of NO-prodrugs (such as organic nitrates and sodium nitroprusside) in the target tissue, uncertainty about which part of NO's action profile contributes to cardioprotection and how it may be influenced by other breakdown products of NO-donor metabolism, and/or undesirable pharmacodynamic effects such as systemic blood pressure reduction and ‘coronary steal’. Some of these limitations and uncertainties would seem to be non-existent when gaseous NO rather than an NO-donor was used for preconditioning.

Inhaled nitric oxide (iNO) is a selective pulmonary vasodilator commonly used to treat neonatal hypoxia and pulmonary hypertension [17]. Although it does not affect systemic blood pressure, many extra-pulmonary benefits of NO inhalation have been described. In animal models, breathing NO reduces cardiac [[18], [19], [20], [21]], intestinal [22], lung [23], and hepatic I/R injury [24], and increases the survival rate of mice resuscitated after cardiac arrest [25]. In humans, iNO reduces inflammatory responses following tourniquet-induced lower extremity I/R injury in patients undergoing elective knee surgeries [26], and decreases hepatocyte apoptosis after liver transplantation [27].

Although NO has a short half-life in biological fluids [28], prolonged effects of iNO have been reported [29]. For example, in mice and sheep, pre-treatment with iNO prevented systemic vasoconstriction induced by the subsequent administration of hemoglobin-based oxygen carriers. After discontinuation of NO breathing, inhibition of vasoconstriction persisted for 80 min in mice and up to 5 h in sheep [29]. Free NO cannot account for these sustained effects in blood *in vivo* as it is rapidly scavenged by hemoglobin in erythrocytes [30]. However, NO can be transported in bioactive form in blood [31,32]. In human studies, breathing NO increases erythrocyte nitrosyl-hemoglobin (NO-heme) [33] and NO_x_ (the sum of nitrite and nitrate) [34] concentrations. These NO metabolites have considerably longer half-lives than NO itself. In erythrocytes, for example, the half-lives of NO-heme and NO_x_ are 40 min [33] and 180 min [34], respectively. In peripheral tissues, circulating NO metabolites may be converted back to NO to exert local protective effects [35], thereby allowing beneficial effects of iNO to persist after cessation of inhalation. However, the fate of these metabolites in blood and tissues has not been studied in much detail. Apart from differences in absolute levels at any one time, it is also unknown how well changes in circulating concentrations of NO metabolites may reflect those occurring in tissues, and whether the lifetimes of individual NO metabolite classes differ between organs.

We previously reported that breathing NO reduces cardiac ischemia-reperfusion (I/R) injury in animal models [[18], [19], [20], [21]], that increased levels of NO metabolites in blood and tissues correlate with the cardioprotective effects of iNO [20], and that these effects are dependent on the presence of soluble guanylate cyclase [21]. In these studies, NO was administered during the ischemic phase and maintained throughout some or all of the reperfusion; thus, NO may have acted as a peri- or post-conditioning agent [36,37]. In the present study we hypothesized that increases in blood and tissue NO metabolites following NO inhalation given *before* ischemia may persist long enough to confer protection during the most critical time for induction of I/R injury (the first few minutes of reperfusion [38]), and through part of the recovery period. Breathing NO prior to cardiac ischemia may produce an increase in NO metabolites, such as S-nitrosothiols (RSNO) [39], that persists throughout the ischemic and early reperfusion periods. These NO metabolites may induce NO's second messenger, cyclic guanosine monophosphate (cGMP) [40,41] in the heart, which may afford cardiac protection via pharmacological pre-conditioning for an as yet undefined period of time afterwards.

We here report that pre-treatment with iNO before induction of ischemia increases cardiac and blood NO metabolites to a level sufficient to reduce cardiac I/R injury in mice. Since similar increases in NO metabolite concentrations were observed in other tissues NO inhalation may also protect organs other than the heart in the peri-operative setting. Our study is timely inasmuch as results from NOMI, the largest double-blind randomized controlled trial on NO inhalation in patients with ST-elevation myocardial infarction to date, have just been published [42]. While iNO (80 ppm, given before PCI and up to 4 h after reperfusion) was found to be safe, it did not reduce infarct size. At 4 months, an improved recovery of left ventricular function was apparent with iNO, but for the primary end point the combination of nitroglycerin (a peri-procedural medication that is part of routine clinical practice in many countries) and iNO showed a trend towards harm [42]. The reason for this unfavorable effect is unknown, but similar interactions may have contributed to the disappointing results with nitrite as peri-conditioning agent in two other recent clinical trials [43,44]. The editorial accompanying publication of the NOMI trial results suggested that it may be time to take a reverse translational approach and study in more detail optimal dose and timing of iNO initiation [45]. Our present investigation would seem to be the first to contribute toward closing the existing knowledge gap concerning the lifetimes of cardiac NO metabolites after NO inhalation and the heterogeneity in metabolic profiles and lifetimes in other organs.

## 2 Materials and methods

### 2.1 Mice

Ten to twelve week-old male C57BL/6J mice on a standard diet (RMH 3000, Prolab; PMI International, St. Louis, MO) were used for this study and maintained in a temperature and humidity controlled environment at a regular 12:12 h light/dark cycle. All experimental protocols were approved by the Subcommittee on Research Animal Care of Massachusetts General Hospital, Boston, MA, USA, and the Institutional Animal Care and Use Committee at University of Warwick, Warwick Medical School, Coventry, UK.

### 2.2 Decay of NO metabolites in mice breathing NO

We measured NO metabolites in blood and tissues of mice that breathed 80 parts per million (ppm) NO for 1 h to study their decay profile after NO exposure. These conditions were chosen based on results from our previous study demonstrating that steady-state levels were reached for most NO-metabolites after ∼60 min at this concentration of NO [20]. Mice were placed in a chamber (PLY3211; Buxco Research Systems, Wilmington, NC) and exposed for 60 min to medical-grade air supplemented with 80 ppm NO, achieved by mixing 10,000 ppm NO in nitrogen (Medical-Technical Gases, Medford, MA) with medical-grade air (Airgas East, Salem, NH). Mice were anesthetized with diethylether and euthanized just before or 10, 30, 60, 180, 720, 1440 and 2880 min after breathing NO (n = 4–8 mice at each time point: Supplementary Fig. 1). Control mice breathed medical-grade air in the chamber for 60 min (n = 4–5: Supplementary Fig. 1). Following thoracotomy, blood was withdrawn from the left ventricle (LV) by cardiac puncture using an EDTA-containing syringe and immediately centrifuged (2800×*g*, 10 min, 22 °C) to separate blood cells from plasma. Erythrocytes (cell pellet after removal of buffy coat) were subjected to hypotonic lysis in distilled water supplemented with N-ethylmaleimide and EDTA (10 mM/2.5 mM). A whole-body exchange of blood against buffer was performed by perfusing tissues for 60 s via the LV with room air-equilibrated phosphate-buffered saline supplemented with N-ethylmaleimide (10 mM) and EDTA (2.5 mM). Heart, brain, lungs, liver, and kidneys were excised, in that order, quickly blotted dry on filter paper, and weighed; urine was collected by bladder puncture. All biospecimen were snap frozen in liquid nitrogen immediately after collection/processing and kept frozen at −80**°**C for a maximum of 2 months before being courier shipped on dry ice for analysis.

### 2.3 Measurement of NO metabolites

Nitrite, nitrate, S-nitrosothiol (RSNO), N-nitrosamine (RNNO), and nitrosylheme (NO–heme) species were quantified in blood (plasma and lysed erythrocytes) and organ homogenates (heart, brain, lungs, liver, and kidney). Blood samples were thawed on ice, vortexed and analyzed immediately. Frozen tissues were thawed in N-ethylmaleimide/EDTA (10 mM/2.5 mM)-supplemented phosphate buffered saline at a ratio of 1:4 (weight:volume) and homogenized using a Potter Elvehjem-type tissue grinder (glass/glass for aorta, PTFE/glass for all other tissues); samples were processed sequentially and subjected to immediate analysis to minimize batch effects. Due to sample volume limitations, plasma analysis was restricted to nitrite, nitrate, and RxNO (sum of RSNO + RNNO), while urine analysis was restricted to nitrite and nitrate measurements. Methods for the detection of nitrosation (RSNO and RNNO) and nitrosylation (NO–heme) products, as well as the oxidation products of NO (nitrite and nitrate), in blood and tissues were described previously [46]. Briefly, quantification was achieved by group-specific denitros(yl)ation after injection of samples into either a triiodide-containing reaction mixture (for the determination of nitrosated products) or a potassium ferricyanide solution (for the determination of nitrosylated products) constantly purged with argon, with NO detection by gas phase chemiluminescence (CLD 77am sp; EcoPhysics Ann Arbor, MI). Oxidation products of NO were quantified by high pressure liquid chromatography using a dedicated analysis system (ENO20 with Gilson autosampler and EDAQ/PowerChrom for data processing; Eicom, San Diego, CA).

### 2.4 Pilot studies

A series of small pilot investigations was conducted in C57BL/6J mice breathing either medical-grade air or 80 ppm NO in air for 1 h while freely moving in acrylic chambers to establish the feasibility of measuring NO metabolite profiles in blood and tissues using frozen samples. We were concerned that when analyzing frozen samples the concentrations of metastable NO metabolites obtained might be orders of magnitude lower compared to fresh samples, potentially compromising experiments aimed at studying NO metabolite lifetimes. A second aim of these pilot experiments was to gain preliminary insight into the lifetime of tissue NO products for up to 3 h after NO inhalation. While the NO metabolite profile after 1 h of iNO administration was similar to that observed in our previous study [20], measured concentrations were different for some, but not all metabolite classes, particularly in the control group. Apart from alterations induced by the freeze/thaw process those differences may be attributable to diurnal and seasonal variation, litter, and variations in dietary NO_x_ content of tap water and rodent chow (consistent with earlier observations in Wistar rats; Feelisch et al., unpublished data). Since neither of these factors had been controlled for in these orientating studies, we opted not to include this data in our main study.

### 2.5 Cardiac ischemia-reperfusion injury

Mice (n = 10 and 8 for control and iNO, respectively) were anesthetized by intraperitoneal administration of ketamine (120 mg/kg) and xylazine (5 mg/kg). After endotracheal intubation, mice were ventilated using a MiniVent 845 (Hugo Sachs Elektronik-Harvard Apparatus GmbH; March-Hugstetten, Germany). Cardiac I/R injury was induced by ligation of the left anterior descending (LAD) coronary artery for 60 min, followed by removal of the occlusion and reperfusion. After 24 h, the LAD coronary artery was re-ligated, and fluorescent microspheres (0.25 ml, 10-μm diameter, FluoSpheres; Invitrogen Corporation, Carlsbad, CA) were injected into the left ventricle (LV) to determine the area at risk (AAR). The heart was excised, and four consecutive 1-mm thick cardiac sections were stained with 2,3,5-triphenyltetrazolium chloride (1% wt/vol; Sigma-Aldrich, St. Louis, MO) for the measurement of myocardial infarction (MI) size. LV, AAR, and MI areas were measured by computer-assisted planimetry (NIH Image J 1.34; Bethesda, MD), and AAR/LV and MI/AAR ratios were calculated as previously described [20]. Mice in the NO inhalation group received 80 ppm NO for 40 min diluted in medical-grade air in vented acrylic chambers [47]. After anesthesia induction, mice were ventilated with a FiO_2_ near 1.0 and 80 ppm NO for an additional 20 min until the coronary artery was exposed and two 7-0 suture silks were passed beneath it. NO exposure was discontinued when the coronary artery was ligated. Control mice breathed medical grade air for 40 min (in the aforementioned acrylic chamber) and 100% O_2_ during the procedure. The acrylic chambers held atmospheres with stable FiO_2_, NO_2_ (nitrogen dioxide) and NO levels. The levels of O_2_ and NO_2_ were measured continuously with the iNOVent delivery system (Ikaria, Hampton, NJ), and the NO concentration was monitored by gas-phase chemiluminescence (Sievers NOA 280; GE Analytical Instruments, Boulder, CO).

### 2.6 Statistical analysis and half-life determination

Each NO metabolite level in mice breathing NO was compared with its mean baseline level in control mice breathing air using the Mann-Whitney-U test. Data are presented as mean values ± standard error (SEM). No adjustment for multiple comparisons was made; p values < 0.05 were considered significant.

In initial attempts to determine the half-life of NO metabolites we used non-linear pharmacokinetic regression analysis. Some NO metabolites (such as the erythrocytic NO-hemoglobin decay in blood) showed a monophasic decomposition profile that could be readily fitted to an exponential decay, from time point zero after breathing 80 ppm NO for 1 h to the point at which baseline levels were reached again. However, most other decay profiles were considerably more complex. For monophasic exponential decay profiles calculated half-lives were in excellent agreement with data obtained by graphical extrapolation, but marked inconsistencies were apparent for analytes showing more complex behavior. For multi-phasic curves, we initially applied separate regression analysis for the different phases of the curve to which we added the time to reach peak levels, where appropriate; however, outliers tended to have a large impact on these calculations. Due to the moderate number of animals per individual time points and some missing data (sample loss due to thawing during one shipment) we opted not to eliminate outliers to avoid bias. For sake of consistency, all NO metabolite half-lives were therefore determined manually from the graphs. ‘Half-life’ was defined as the time window when concentrations reached 50% of peak concentrations achieved for a particular analyte. In most cases this corresponded to 0 min, i.e. the time directly after cessation of NO inhalation, but maximal levels of hepatic nitroso species, for example, were observed considerably later. All calculations and statistical analyses were performed using GraphPad Prism (La Jolla, CA).

## 3 Results

### 3.1 Feasibility studies establish that even labile NO metabolites can be analyzed in frozen samples

Since all samples of our earlier murine study on the metabolic fate of iNO [20] had been analyzed within minutes of specimen harvest there was a need to establish whether or not frozen blood/tissue samples could be used to study NO metabolite decay. While this is firmly established for nitrite/nitrate analysis little information is available for more labile NO adducts such as NO-heme species. We found that the pattern of NO metabolites arising (little or no increase in nitrite, marked elevations in nitrate, measurable elevations in RSNO and NO-heme species and a lack of increase in cardiac RNNOs) after 60 min of breathing 80 ppm NO reported earlier [20] was faithfully reproduced. For some compartments and analyte classes absolute values in frozen tissue were somewhat lower after iNO compared to published values, whereas the opposite trend was observed in the control group exposed to air. However, since the magnitude of increases in NO-metabolite concentrations 1 h after NO inhalation was not too dissimilar from that in our previous study [20] we reasoned that frozen biospecimen can be used to gain insight into the *in vivo* lifetime of NO metabolites. Our pilot studies further suggested that the time course of investigation required extension well beyond 3 h.

### 3.2 Blood and tissue NO metabolites remain elevated for considerable time after breathing NO

To identify key NO metabolites potentially responsible for prolonged beneficial effects of pre-treatment with iNO, we next examined the metabolic fate of NO absorbed during inhalation using more stringent experimental conditions for a maximum of two days after NO inhalation. Mice that were treated with 80 ppm NO for 60 min or control animals were anesthetized and sacrificed at defined time points between 0 and 48 h, and levels of NO metabolites (nitrite, nitrate, RSNO, RNNO, and NO-heme) in blood and tissues were measured. Results for erythrocytes and plasma are depicted in Fig. 1A and Supplementary Table 1; those for heart, lung, liver, kidney and brain are displayed in Fig. 1B and Supplementary Tables 2-4. In addition, nitrite and nitrate concentrations were measured in urine contained in the bladder at autopsy (see Supplemental Table 4 and Supplementary Fig. 2). The main observations from these studies are summarized by compartments below.

**Fig. 1A:**
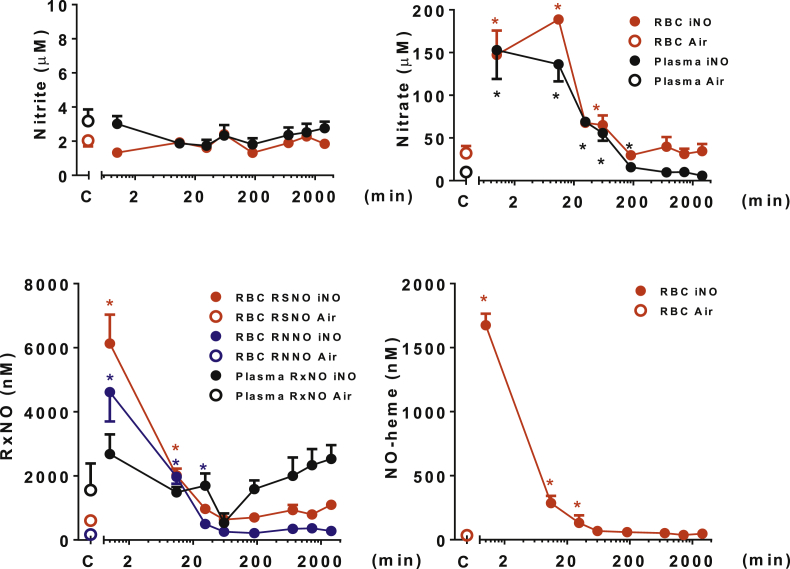
**Distribution and kinetics of NO metabolites in blood (erythrocytes and plasma)**. Concentrations of NO metabolites were measured in the blood of mice breathing air with 80 ppm NO for 60 min, harvested at 0, 10, 30 min, 1, 3, 6, 12, 24 and 48 h (NO, n = 4–7). Control mice breathed air for 60 min (n = 4–5). *P < 0.05 versus mice not breathing NO. C = control group. NO-heme = nitrosyl-heme species; RBC = erythrocytes; RNNO = N-nitrosamines; RSNO = S-nitrosothiols. Additional information regarding each concentration, the number of animals studied, and the P value are available in the Supplemental Table 1.

**Fig. 1B:**
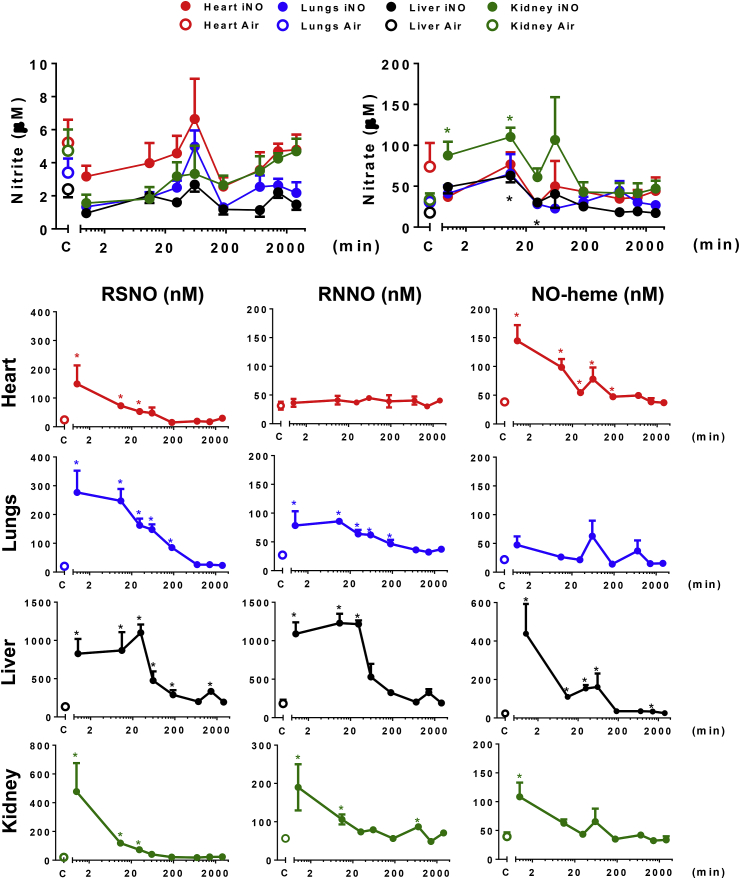
**Distribution and kinetics of NO metabolites in the tissues (heart, lungs liver and kidney)**. Distribution and kinetics of accumulation of NO metabolites in mice breathing NO (heart, lungs, liver and kidney). Concentrations of NO metabolites were measured in tissues of mice breathing air with 80 ppm NO for 60 min, harvested at 0, 10, 30 min, 1, 3, 6, 12, 24 and 48 h (NO, n = 3–7). Control mice breathed air for 60 min (n = 4–5). *P < 0.05 versus mice not breathing NO. C = control group. NO-heme = nitrosyl-heme species; RNNO = N-nitrosamines; RSNO = S-nitrosothiols. Additional information regarding each concentration, the number of animals studied, and the P value are available in the Supplemental Tables 2-4.

#### 3.2.1 NO metabolite changes in blood

After breathing NO for 60 min, nitrite levels in blood did not differ significantly from baseline. This is in contrast to an earlier investigation [18], but consistent with our previous study in mice [20]. In contrast, breathing NO for 60 min increased many other NO metabolites in the blood.

Nitrate, RSNO, RNNO and NO-heme concentrations were all increased in erythrocytes from NO-treated mice compared with control animals. Nitrate, but not RxNO levels were also increased in plasma from NO-treated mice compared with plasma from control mice; plasma NO-heme levels were below the detection limit in both treatment groups. After discontinuing NO inhalation, erythrocytic nitrate, RSNO, RNNO and NO-heme concentrations decreased to baseline levels within 10–30 min (Fig. 1A, Supplementary Table 1). Plasma nitrate remained significantly elevated for up to 180 min (Fig. 1A and Supplementary Table 1). In summary, changes in nitrate concentrations were similar in plasma and red blood cells, and both, nitroso and nitrosyl species elevations in erythrocytes were short-lived.

#### 3.2.2 NO metabolite changes in tissues

*Heart:* Since we wanted to learn whether breathing NO before subjecting the heart to ischemia protects against myocardial reperfusion injury the changes of NO metabolites in cardiac tissue were of particular interest. Breathing NO for 60 min increased RSNO and NO-heme levels in the heart, and these levels remained elevated for 30 and 180 min, respectively, when compared to baseline (Fig. 1B and Supplementary Table 2). The levels of nitrite, nitrate and RNNO in the heart did not differ at any time from baseline levels.

*Lungs*: Breathing NO increased RSNO and RNNO levels in the lungs without altering NO-heme concentrations. Sustained increases of RSNO and RNNO levels were detected for up to 180 min after cessation of NO breathing, with gradual decreases thereafter (Fig. 1B, Supplementary Table 2). Despite the lungs being exposed to the highest NO concentrations, no significant changes in nitrite or nitrate were apparent.

*Liver*: The liver displayed the highest levels of nitroso and nitrosyl products compared with all the other tissues we studied. Hepatic RSNO and RNNO levels exceeded 1 μM (Fig. 1B and Supplementary Table 3) and remained elevated at an almost constant level for about 30 min after discontinuation of NO breathing before dropping in an exponential manner. In contrast to the levels of RSNO and RNNO, hepatic NO-heme decreased rapidly upon cessation of NO exposure (Fig. 1B, Supplementary Table 3). Nitrate, but not nitrite, was modestly elevated 10 and 30 min after NO inhalation.

*Kidneys*: In the kidneys, elevated RSNO, RNNO and NO-heme levels began decreasing immediately after cessation of NO inhalation, whereas kidney nitrate remained elevated for 10 min and then decreased rapidly (Fig. 1B, Supplementary Table 3). As in the other organs, considerable fluctuation in nitrite and nitrate concentrations occurred in the first 2 h after NO exposure.

*Brain*: In contrast to the other tissues we studied, breathing NO did not increase the levels of any NO metabolites in the brain (Supplementary Table 4).

Thus overall, NO metabolite elevations persisted longer in tissues than in blood, and decay profiles were organ-specific. Nitrite did not increase in blood or tissues with NO inhalation; if anything, levels tended to be lower than in the controls right after cessation of NO inhalation. However, considerable fluctuations around the baseline levels were seen for both nitrite and nitrate 20–60 min after NO exposure. Peak levels of most of the other NO metabolites were observed immediately after cessation of NO inhalation, and shortly thereafter either decreasing immediately or remaining elevated for variable periods of time before decreasing rapidly (Fig. 1B, Fig. 1AA and B, Supplementary Tables 1-4).

#### 3.2.3 Excretion of nitrite and nitrate in urine

Consistent with the notion that nitrate is renally excreted as the endproduct of oxidative NO metabolism, breathing NO markedly increased urinary nitrate but did not alter urinary nitrite concentrations significantly (Supplementary Table 4 and Supplementary Fig. 2).

### 3.3 The half-life of NO metabolites in blood differs from that in tissues

The lifetimes of NO metabolites in blood did not reflect those in tissues, and the characteristics of tissue NO metabolites appear to differ from organ to organ. Moreover, apparent lifetimes vary between compound classes. In some cases (e.g. RNNO in heart and nitrite throughout all compartments), their lifetime could not be established because steady-state concentrations did not change with NO inhalation. Erythrocytic NO metabolites presented with shorter half-lives (<10 min for nitrate, RSNO, RNNO and NO-heme) than cardiac RSNOs and NO-hemes (10–15 min), whereas NO metabolites in liver spanned from 3 min (NO-heme) to approximately 40 min (RSNO, RNNO); the latter is largely due to the fact that for about 20–30 min steady-state concentrations in hepatic tissue did not change despite cessation of NO inhalation. Except for nitrate (70 min), the half-lives of NO metabolites in the kidney were all rather short (RSNO, RNNO and NO-heme: 5–15 min). Comparing the lifetimes of NO metabolites by class and organ system (Supplementary Fig. 3) did not reveal any obvious pattern that would allow us to infer a pecking order of stabilities and associated NO-release potential and explain in what form NO equivalents are transported from the lung to individual organs and perhaps exchanged between cells and tissues.

### 3.4 Pretreatment with inhaled NO attenuates cardiac ischemia-reperfusion injury

**Fig. 2:**
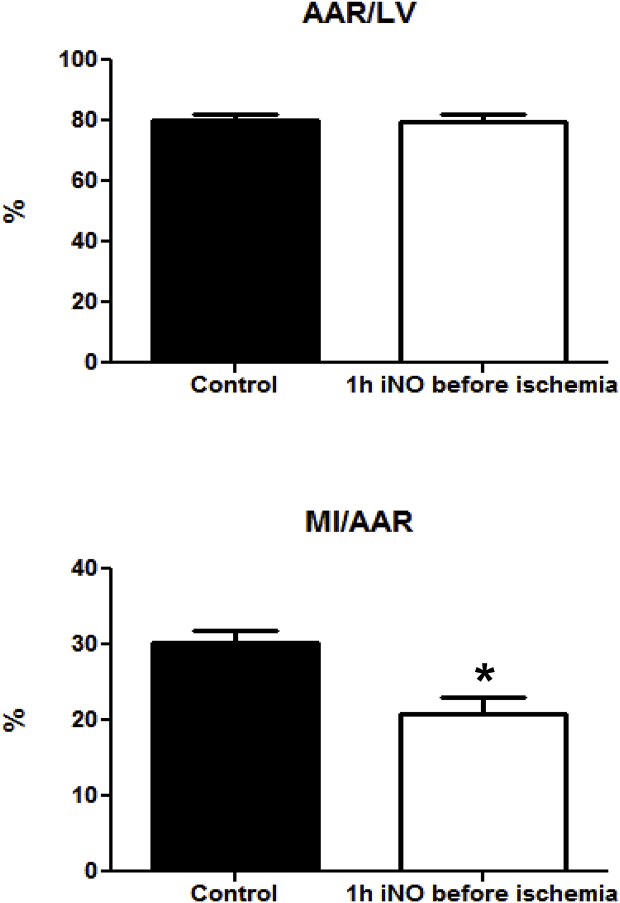
**Pretreatment of NO inhalation limits myocardial ischemia–reperfusion injury.** All mice underwent left coronary artery occlusion for 60 min followed by 24 h of reperfusion. Mice received 80 ppm NO, prior to coronary artery occlusion for 60 min (n = 10 and 8 for control and inhaled NO, respectively). Control mice did not receive NO. Breathing NO decreased MI/AAR by 30% (*P = 0.002 versus control, lower panel). AAR/LV did not differ between two groups (upper panel). AAR = area at risk; LV = left ventricle; MI = myocardial infarction.

Previous studies demonstrated that breathing NO reduces cardiac I/R injury in mice [18,20,21] and swine [19]. In the current study, we sought to test whether breathing NO was also protective when administered during the 1 h preceding initiation of cardiac ischemia. We found that the MI/AAR ratio was reduced by 30% in mice pretreated with 80 ppm NO for 60 min as compared to mice breathing only air (30 ± 1% without NO vs. 21 ± 2 with NO, p = 0.002; Fig. 2, lower panel). The AAR/LV ratio, a measure of the left ventricular area subjected to ischemia, did not differ between mice breathing air/oxygen without or with 80 ppm NO (80 ± 2 vs. 79 ± 3%, respectively, p = n.s.; Fig. 2, upper panel). Thus both groups had equal areas subjected to ischemia by coronary artery ligation. These results strongly support the notion that tissue storage forms of NO can confer cardioprotection and iNO can act as a preconditioning agent.

## 4 Discussion

In awake mice that were not subjected to cardiac injury, 1 h of NO inhalation resulted in prolonged increases of various NO metabolites in blood and tissues. Alterations in NO metabolites were non-uniform, with considerable variation in the lifetime of distinct species within one compartment and marked differences between organs. Changes in plasma and erythrocytes did not reflect the status of any of the tissue metabolites, potentially limiting the value of blood-based biomarkers to gauge NO-related processes in organs. NO metabolite elevations in the heart, in particular in the form of NO-heme products, may persist throughout the immediate reperfusion period and contribute to the cardioprotective effects of iNO by serving as sources of NO in the tissue area at risk. As a proof-of-principle we here demonstrate that a 1-h pre-treatment with 80 ppm NO of mice that were subsequently subjected to myocardial I/R injury decreases infarct size. These findings extend previous experimental results on the protection of lung tissue in a porcine model [48,49] to the heart and firmly establish short periods of NO breathing as a viable pharmacological preconditioning modality.

Dysfunctional NO signaling has been shown to increase the risk of myocardial infarction [50]. Because NO itself is a short-lived molecule in the body, increased levels of NO metabolites in blood and tissue may directly or indirectly protect the heart in the setting of cardiac I/R injury [51]. We previously established that inhalation with 80 ppm NO leads to rapid and widespread increases in the concentration of multiple NO metabolites, with considerable differences in maximal concentrations achieved in different cells/organs, and that breathing NO for as short as 5 and 60 min confers cardioprotection [20]. In this earlier study, NO was administered during the ischemic phase just before reperfusion. It therefore remains unclear whether the protective effect was conferred by NO-containing material circulating in blood or resident in cardiac tissue. However, due to the vascular occlusion used to produce cardiac ischemia in this model, iNO could not have reached myocardial tissue at risk via the blood; this would seem to exclude the possibility that NO could have acted as a preconditioning agent in this instance. In which particular form NO is transported following inhalation by the lungs to peripheral organs remains a matter of speculation at present. Clearly, it involves circulating adducts/storage forms; NO appears to be “hitchhiking” on albumin in plasma and on hemoglobin in red blood cells [31,32], but we cannot exclude the involvement of other blood elements at this stage. Conceivably, once elevated some of the tissue metabolites may serve as long-acting storage forms of NO [35] that can confer protection against injury at a later stage, either by directly releasing NO (from RSNO and NO-heme species) or following metabolic conversion to NO (from nitrate, nitrite and RNNO). This NO may then stimulate the cGMP-protein kinase G axis, which has been implicated in both ischemic pre- and postconditioning [52]. The two main objectives of the present investigation were to i) gain insight into the half-life of NO-containing species in the heart and other organs (the half-life of NO-related compounds should be a reasonable measure of its ability to serve as a source of NO) and ii) to establish that breathing NO can also be used to precondition the heart against I/R damage.

Administration of NO for the 60 min preceding the initiation of ischemia reduced MI/AAR in our study by 30%. These results are consistent with previous reports that breathing NO reduces cardiac damage in this murine model of I/R injury [20,21]. The present report differs from these earlier studies in that it examined the effects of giving NO as a pre-treatment, before ischemia, to examine the prolonged effects of breathing NO. Our results indicate that, in the setting of patients suffering from unstable angina, pre-treatment with NO inhalation prior to possible progression and complete coronary occlusion may reduce I/R injury.

Pretreatment with iNO may limit myocardial RI by enabling NO metabolites present in the at-risk portion of the heart, formed prior to induction of ischemia, to protect the heart from damage. Which of the NO metabolites generated contributes to protection remains unclear at this stage. In the current study, cardiac RSNO levels were elevated for 30 min after cessation of NO breathing. Increased levels of cardiac RSNO have been shown to confer protection against I/R injury in mice [53]. RSNO may protect the heart by shielding cysteine residues from oxidation by reactive oxygen species during I/R [39]. For example, S-nitrosation of the cysteine-81 residue in macrophage migration inhibitory factor has been shown to decrease apoptosis of reperfused cardiomyocytes [54]; moreover, S-nitrosation of cysteine-69 in human thioredoxin potentiated its antiapoptotic and cardioprotective effects in mice *in vivo* [55]. Breathing NO for 1 h by mice not subjected to cardiac injury increased cardiac NO-heme and plasma nitrate levels even longer (>60 min after NO breathing was discontinued). These results suggest that in this murine model of cardiac I/R injury, NO metabolites are present during the reperfusion period and may also exert a cardioprotective role via a post-conditioning process [36]. It is conceivable that protection is afforded not by a single NO-containing product in the target tissue but by a combination of metabolites and cell types including activated platelets and neutrophils, which are known to contribute to myocardial RI [56,57] but have not been specifically investigated in the present study.

The rate of decay of tissue NO metabolites was organ-specific and of considerably greater complexity than anticipated. This inter-compartmental heterogeneity and the complexity of the dynamics suggest either differential regulation, differences in chemical nature and/or stability, inter-organ transfer of NO equivalents, or a combination of those factors. In the lungs, RSNO and RNNO were elevated up to 180 min after NO breathing without changes in NO-heme levels, whereas in the heart only RSNO and NO-heme levels were elevated. Consistent with our previous report [20], brain NO metabolite levels did not increase at all after breathing NO, suggesting that NO metabolites either do not cross the blood-brain barrier or their concentrations in neuronal tissue are kept constant. Similarly, cardiac RNNO and nitrite levels did not change with NO inhalation indicating their concentrations are actively regulated. The lack of nitrite increases in blood and tissues is noteworthy and would seem to exclude this NO metabolite as a major contributor to tissue protection when iNO is used as a preconditioning agent. While this observation is consistent with our own earlier results [20] and most human studies in which circulating nitrite increases with 80 ppm NO are typically on the order of 10–20% [42,58] there are two notable exceptions: one relates to a randomized controlled trial of iNO in human liver transplantation [59], the other to the use of an even lower concentration (20 ppm) of iNO in newborn babies [60] in which circulating nitrite levels rose 3 and 4-fold, respectively. The reasons for this discrepancy are not immediately obvious, may involve multiple factors (including species differences, study design, NO delivery specifics as well as duration and timing of iNO application) and are likely to be complex as the origin of nitrite in the circulation and details of its sensing and regulation remain largely unknown.

Together with the lungs, the liver may serve as a reservoir of NO metabolites, providing beneficial effects of inhaled NO throughout the myocardial reperfusion period. The highest levels of tissue nitrosation (RSNO and RNNO) products (800–1000 nM) were measured in the liver, with increased levels persisting for up to 24 h. Curiously, RSNO and RNNO (but not NO-heme) concentrations remained elevated and steady for a considerable time after NO breathing. Whether this is as result of inhibition of metabolizing enzymes (denitrosylases) or due to the efficient capture of NO equivalents by the liver during perfusion with blood is unknown. The liver synthesizes a variety of proteins (albumin and biologic amines) that may serve as carriers of NO metabolites. For example, oxidation of NO in the albumin hydrophobic core and the transfer of NO^+^ to low-molecular weight thiols may allow formation of low-molecular-weight S-nitrosothiols (RSNO) [61]. Thus, the liver may serve as a NO storage pool, and proteins produced by the liver may serve as shuttles for inter-organ transport of NO from storage sites to peripheral organs, such as the heart. Testing the validity of these assumptions will require the use of stable isotope (^15^N) tracer experiments; this was well beyond the purpose of the current study.

Our study has strengths and limitations. Its strengths relate to the controlled administration of authentic NO rather than an NO-donor to study the *in vivo* lifetime of several classes of NO metabolites in blood and multiple organs. To our knowledge, the present study is the most detailed investigation on this matter to date. However, our study does not shed any new light onto the mechanism by which NO confers cardioprotection and was not powered to determine exact half-lives of the various NO-related products monitored. The latter is also due to the unexpected complexity of metabolic changes observed following NO inhalation. Moreover, the experimental conditions used to determine NO metabolite lifetimes were not directly comparable to those used in the MI studies. Cardiac ischemia may alter the fate of NO metabolites in the heart (we have shown 5–7-fold greater levels of cardiac NO-metabolite concentrations while breathing NO in hypoxia compared to normoxia [20]) while hyperoxic conditions (as experienced in the initial phase of reperfusion) may have the opposite effect, further complicating matters and rendering a direct comparison impossible. The cardiac levels of NO metabolites in mice experiencing I/R injury within ischemic myocardium may therefore be even greater than the levels of NO metabolites or prolong the lifetime of selected cardiac NO metabolites that we detected in the uninjured and healthy mice in this study [62].

Translating promising animal experimental results into effective clinical applications has been particularly challenging in the area of myocardial RI; the underlying reasons are multifactorial and have been reviewed elsewhere [4,5,13,16]. In spite of neutral and even negative results in earlier trials using NO-donors, NO continues to represent a highly attractive cardioprotective principle [63,64], not least because NO has the potential to favorably modulate multiple cellular systems (including platelets, neutrophils, cardiomyocytes, fibroblasts, endothelial and smooth muscle cells) and relevant subcellular targets such as mitochondria at the same time. How exactly NO exerts organ protection is currently unknown, but it may well involve a mixture of direct cGMP/PKG-mediated and cGMP-independent myocardial events with additional smooth muscle relaxing, platelet-inhibitory, antiapoptotic, anti-inflammatory and antioxidant effector elements. NO may also confer protection by modulating electron flow in the mitochondrial respiratory chain secondary to S-nitrosation of complex I and interfering with reactive oxygen species production [[65], [66], [67]]. Together, this may translate into reduced myocardial oxygen demand and lower reactive oxygen species production as well as improved coronary blood flow, endothelial and microvascular function. Such effects would seem to be beneficial for patients undergoing orthotopic heart or lung transplantation or cardiac surgery, in particular when combined with right ventricular dysfunction and pulmonary hypertension [68,69].

In summary, we here report that pretreatment with 80 ppm NO for 1 h prior to ischemia ameliorates cardiac I/R injury. After NO inhalation, NO metabolites persist in the blood and tissues. Cardiac RSNO and NO-heme and plasma nitrate levels remained elevated for 30, 180 and 180 min, respectively. Each of these metabolites can be converted back to NO and thus may serve as NO reservoirs. NO metabolites may act as both pre- and post-pharmacological conditioning agents, explaining the cardioprotection afforded by iNO. Our results provide a framework for novel applications of iNO to prevent cardiac I/R injury. Future efforts should focus on further optimizing the current NO inhalation protocol to establish whether even shorter breathing times may suffice, move the NO application window further ahead to explore boundaries and windows of opportunity for cardioprotection and to test the NO breathing paradigm for prevention of reperfusion injury in other organ systems.

## 5 Conflicts of interest and funding

W. M. Zapol and K. D. Bloch have obtained patents relating to the use of inhaled nitric oxide. These patents are assigned to Massachusetts General Hospital, which has licensed them to Ikaria and Linde Gas Therapeutics (Lidingo, Sweden). W. M. Zapol receives royalties, and K. D. Bloch received research grants from Ikaria. A. U. Steinbicker received a grant from Deutsche Forschungsgemeinschaft (DFG, SW119/3–1). R. Malhotra received support from the National Heart, Lung, and Blood Institute (K08HL111210). K.D. Bloch and D.B. Bloch received support from the Leducq Fondation and from RO1DK082971. M. Feelisch acknowledges support from the UK Medical Research Council (G0701115, G1001536).

## Appendix A Supplementary data

Data Profile Data Profile

Supplementary material Supplementary material

Suppl. Table 1: RBC and Plasma.

Suppl. Table 2: Heart and Lungs.

Suppl. Table 3: Liver and Kidney.

Suppl. Table 4: Brain and Urine.

Blood and tissue analysis of NO metabolites in mice that breathed medical-grade air or air supplemented with 80 ppm NO for one hour. Data are expressed as average ± SEM (number of animal). * P<0.05, value differs vs. control mice breathed air for 60 min (Mann-Whitney test).

Abbreviations: nitric oxide (NO), nitrosyl-heme species (NO-heme), N-nitrosamines (RNNO), S-nitrosothiols (RSNO).

Supplementary Fig. 1: NO metabolites were measured at 0, 10, 30, 60, 180, 720, 1440 and 2880 min after breathing 80 ppm NO for 60 min. Control mice breathed air for 60 min.

Supplementary Fig. 2: Time-dependent urinary excretion of oxidative NO metabolites in mice breathing NO. Nitrite and nitrate concentrations were measured in urine of mice breathing air for 60 min with 80 ppm NO for 60 min, harvested at 0, 10, 30 min, 1, 3, 6, 12, 24 and 48 h (NO, n=4-8). Control mice breathed air for 60 min (n=8). NO-heme = nitrosyl-heme species; RNNO = N-nitrosamines; RSNO = S-nitrosothiols. Additional information regarding each concentration, the number of animals studied, and the P value are available in the Supplemental Table 4.

Supplementary Fig. 3: Comparison of approximate lifetimes of NO products by metabolite class and organ (range in minutes, as extrapolated from the graphs; see Methods for details).
