# Supplementary material for: Pharmacological preconditioning with inhaled nitric oxide (NO): Organ-specific differences in the lifetime of blood and tissue NO metabolites
Source: Nitric Oxide. 2018 Nov 1;80:52–60. doi: 10.1016/j.niox.2018.08.006 (PMC6198794; doi:10.1016/j.niox.2018.08.006)
Supplement: Supplementary material [file mmc2.pptx]

## Slide 1
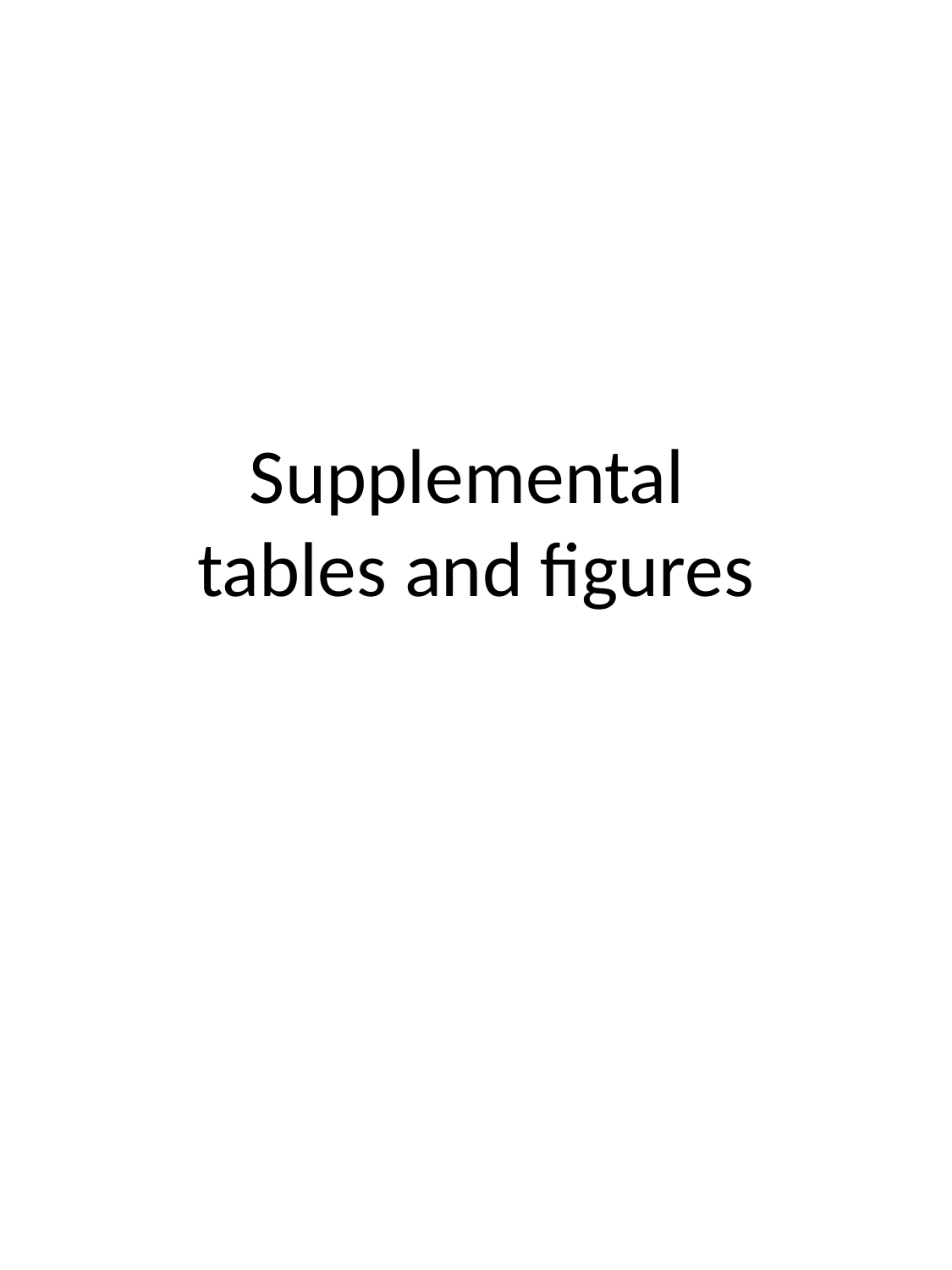

Supplemental
tables and figures

## Slide 2
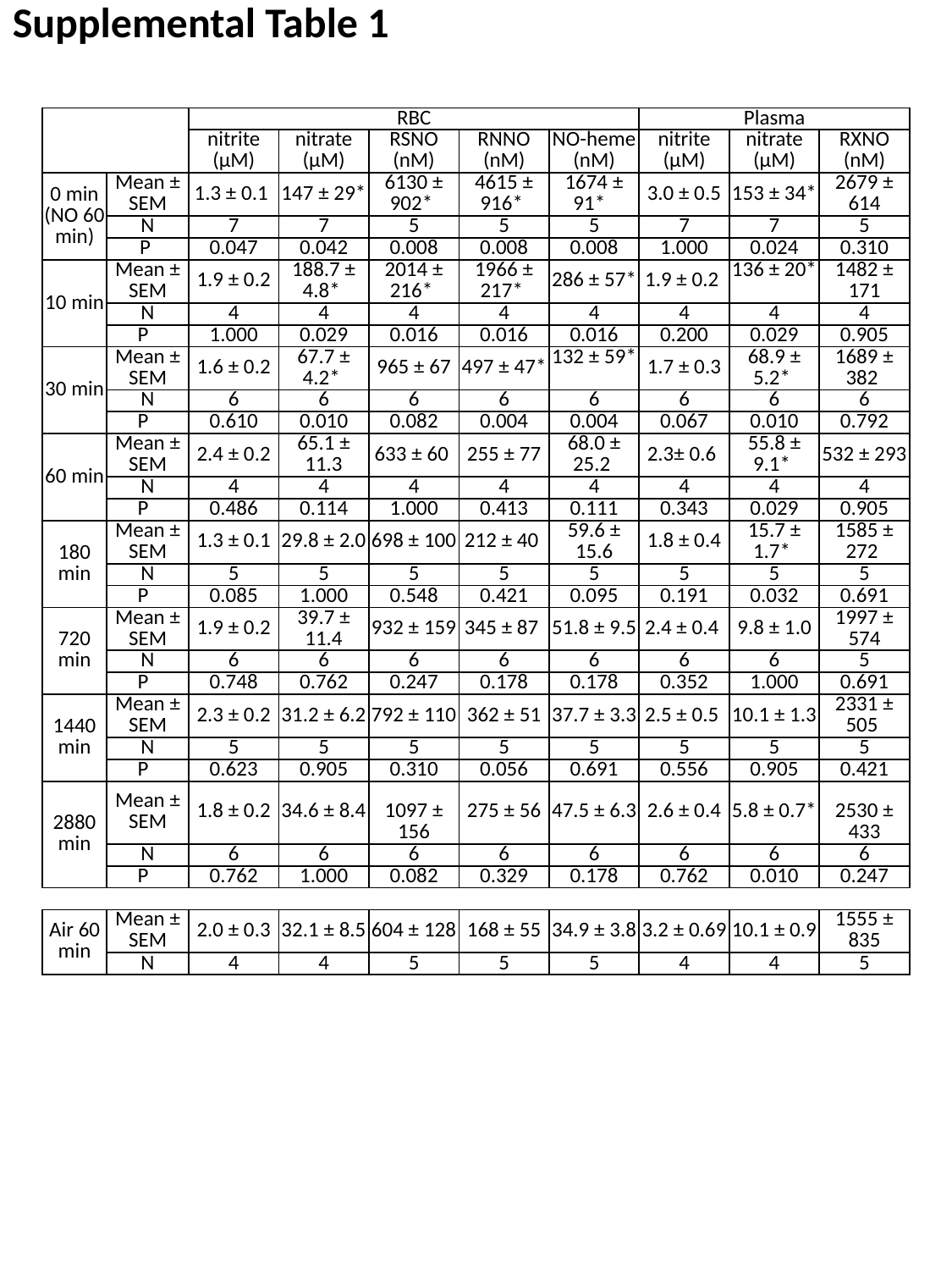

Supplemental Table 1
| | | RBC | | | | | Plasma | | |
| --- | --- | --- | --- | --- | --- | --- | --- | --- | --- |
| | | nitrite (μM) | nitrate (μM) | RSNO (nM) | RNNO (nM) | NO-heme (nM) | nitrite (μM) | nitrate (μM) | RXNO (nM) |
| 0 min (NO 60 min) | Mean ± SEM | 1.3 ± 0.1 | 147 ± 29\* | 6130 ± 902\* | 4615 ± 916\* | 1674 ± 91\* | 3.0 ± 0.5 | 153 ± 34\* | 2679 ± 614 |
| | N | 7 | 7 | 5 | 5 | 5 | 7 | 7 | 5 |
| | P | 0.047 | 0.042 | 0.008 | 0.008 | 0.008 | 1.000 | 0.024 | 0.310 |
| 10 min | Mean ± SEM | 1.9 ± 0.2 | 188.7 ± 4.8\* | 2014 ± 216\* | 1966 ± 217\* | 286 ± 57\* | 1.9 ± 0.2 | 136 ± 20\* | 1482 ± 171 |
| | N | 4 | 4 | 4 | 4 | 4 | 4 | 4 | 4 |
| | P | 1.000 | 0.029 | 0.016 | 0.016 | 0.016 | 0.200 | 0.029 | 0.905 |
| 30 min | Mean ± SEM | 1.6 ± 0.2 | 67.7 ± 4.2\* | 965 ± 67 | 497 ± 47\* | 132 ± 59\* | 1.7 ± 0.3 | 68.9 ± 5.2\* | 1689 ± 382 |
| | N | 6 | 6 | 6 | 6 | 6 | 6 | 6 | 6 |
| | P | 0.610 | 0.010 | 0.082 | 0.004 | 0.004 | 0.067 | 0.010 | 0.792 |
| 60 min | Mean ± SEM | 2.4 ± 0.2 | 65.1 ± 11.3 | 633 ± 60 | 255 ± 77 | 68.0 ± 25.2 | 2.3± 0.6 | 55.8 ± 9.1\* | 532 ± 293 |
| | N | 4 | 4 | 4 | 4 | 4 | 4 | 4 | 4 |
| | P | 0.486 | 0.114 | 1.000 | 0.413 | 0.111 | 0.343 | 0.029 | 0.905 |
| 180 min | Mean ± SEM | 1.3 ± 0.1 | 29.8 ± 2.0 | 698 ± 100 | 212 ± 40 | 59.6 ± 15.6 | 1.8 ± 0.4 | 15.7 ± 1.7\* | 1585 ± 272 |
| | N | 5 | 5 | 5 | 5 | 5 | 5 | 5 | 5 |
| | P | 0.085 | 1.000 | 0.548 | 0.421 | 0.095 | 0.191 | 0.032 | 0.691 |
| 720 min | Mean ± SEM | 1.9 ± 0.2 | 39.7 ± 11.4 | 932 ± 159 | 345 ± 87 | 51.8 ± 9.5 | 2.4 ± 0.4 | 9.8 ± 1.0 | 1997 ± 574 |
| | N | 6 | 6 | 6 | 6 | 6 | 6 | 6 | 5 |
| | P | 0.748 | 0.762 | 0.247 | 0.178 | 0.178 | 0.352 | 1.000 | 0.691 |
| 1440 min | Mean ± SEM | 2.3 ± 0.2 | 31.2 ± 6.2 | 792 ± 110 | 362 ± 51 | 37.7 ± 3.3 | 2.5 ± 0.5 | 10.1 ± 1.3 | 2331 ± 505 |
| | N | 5 | 5 | 5 | 5 | 5 | 5 | 5 | 5 |
| | P | 0.623 | 0.905 | 0.310 | 0.056 | 0.691 | 0.556 | 0.905 | 0.421 |
| 2880 min | Mean ± SEM | 1.8 ± 0.2 | 34.6 ± 8.4 | 1097 ± 156 | 275 ± 56 | 47.5 ± 6.3 | 2.6 ± 0.4 | 5.8 ± 0.7\* | 2530 ± 433 |
| | N | 6 | 6 | 6 | 6 | 6 | 6 | 6 | 6 |
| | P | 0.762 | 1.000 | 0.082 | 0.329 | 0.178 | 0.762 | 0.010 | 0.247 |
| | | | | | | | | | |
| Air 60 min | Mean ± SEM | 2.0 ± 0.3 | 32.1 ± 8.5 | 604 ± 128 | 168 ± 55 | 34.9 ± 3.8 | 3.2 ± 0.69 | 10.1 ± 0.9 | 1555 ± 835 |
| | N | 4 | 4 | 5 | 5 | 5 | 4 | 4 | 5 |

## Slide 3
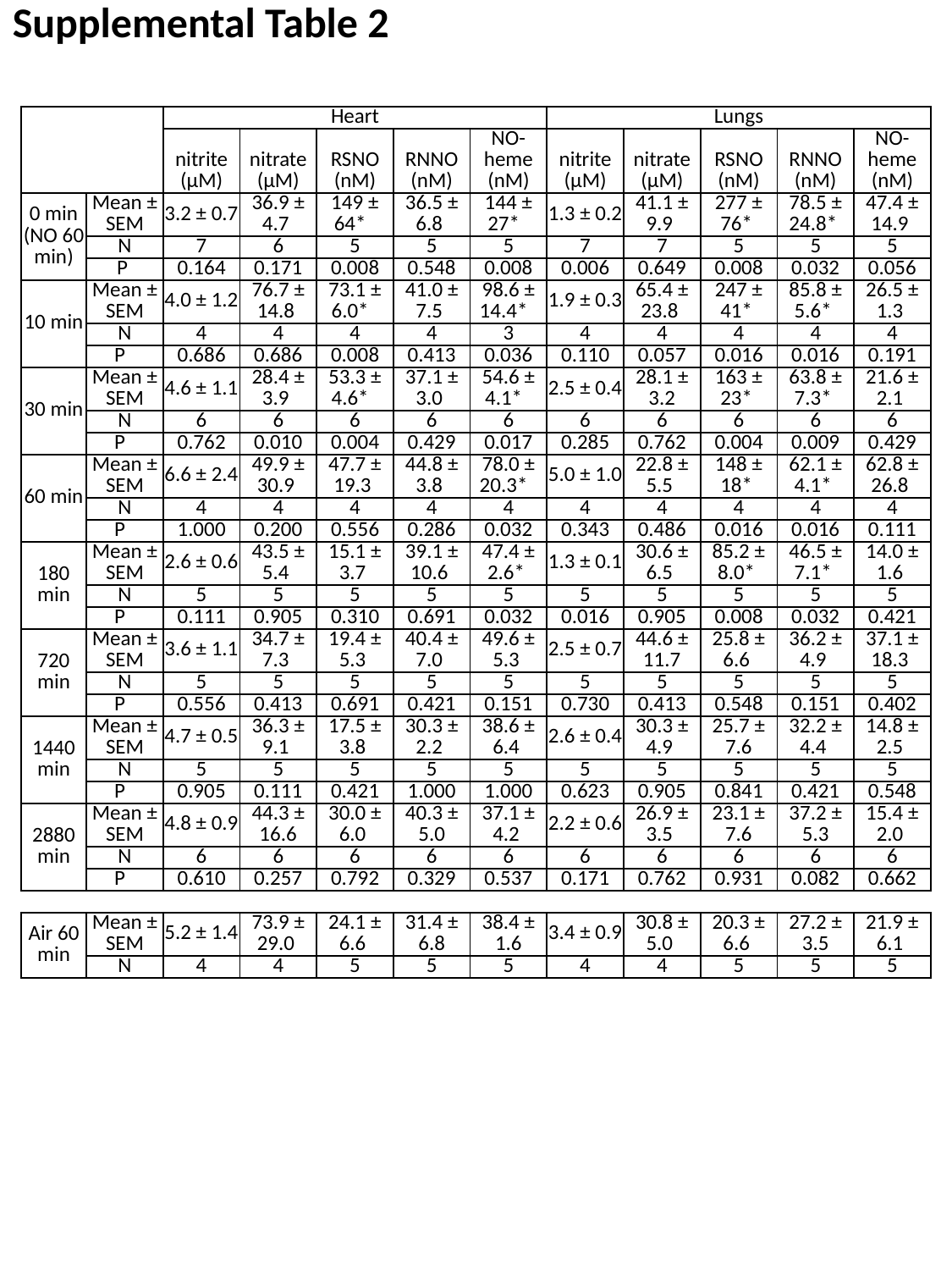

Supplemental Table 2
| | | Heart | | | | | Lungs | | | | |
| --- | --- | --- | --- | --- | --- | --- | --- | --- | --- | --- | --- |
| | | nitrite (μM) | nitrate (μM) | RSNO (nM) | RNNO (nM) | NO-heme (nM) | nitrite (μM) | nitrate (μM) | RSNO (nM) | RNNO (nM) | NO-heme (nM) |
| 0 min (NO 60 min) | Mean ± SEM | 3.2 ± 0.7 | 36.9 ± 4.7 | 149 ± 64\* | 36.5 ± 6.8 | 144 ± 27\* | 1.3 ± 0.2 | 41.1 ± 9.9 | 277 ± 76\* | 78.5 ± 24.8\* | 47.4 ± 14.9 |
| | N | 7 | 6 | 5 | 5 | 5 | 7 | 7 | 5 | 5 | 5 |
| | P | 0.164 | 0.171 | 0.008 | 0.548 | 0.008 | 0.006 | 0.649 | 0.008 | 0.032 | 0.056 |
| 10 min | Mean ± SEM | 4.0 ± 1.2 | 76.7 ± 14.8 | 73.1 ± 6.0\* | 41.0 ± 7.5 | 98.6 ± 14.4\* | 1.9 ± 0.3 | 65.4 ± 23.8 | 247 ± 41\* | 85.8 ± 5.6\* | 26.5 ± 1.3 |
| | N | 4 | 4 | 4 | 4 | 3 | 4 | 4 | 4 | 4 | 4 |
| | P | 0.686 | 0.686 | 0.008 | 0.413 | 0.036 | 0.110 | 0.057 | 0.016 | 0.016 | 0.191 |
| 30 min | Mean ± SEM | 4.6 ± 1.1 | 28.4 ± 3.9 | 53.3 ± 4.6\* | 37.1 ± 3.0 | 54.6 ± 4.1\* | 2.5 ± 0.4 | 28.1 ± 3.2 | 163 ± 23\* | 63.8 ± 7.3\* | 21.6 ± 2.1 |
| | N | 6 | 6 | 6 | 6 | 6 | 6 | 6 | 6 | 6 | 6 |
| | P | 0.762 | 0.010 | 0.004 | 0.429 | 0.017 | 0.285 | 0.762 | 0.004 | 0.009 | 0.429 |
| 60 min | Mean ± SEM | 6.6 ± 2.4 | 49.9 ± 30.9 | 47.7 ± 19.3 | 44.8 ± 3.8 | 78.0 ± 20.3\* | 5.0 ± 1.0 | 22.8 ± 5.5 | 148 ± 18\* | 62.1 ± 4.1\* | 62.8 ± 26.8 |
| | N | 4 | 4 | 4 | 4 | 4 | 4 | 4 | 4 | 4 | 4 |
| | P | 1.000 | 0.200 | 0.556 | 0.286 | 0.032 | 0.343 | 0.486 | 0.016 | 0.016 | 0.111 |
| 180 min | Mean ± SEM | 2.6 ± 0.6 | 43.5 ± 5.4 | 15.1 ± 3.7 | 39.1 ± 10.6 | 47.4 ± 2.6\* | 1.3 ± 0.1 | 30.6 ± 6.5 | 85.2 ± 8.0\* | 46.5 ± 7.1\* | 14.0 ± 1.6 |
| | N | 5 | 5 | 5 | 5 | 5 | 5 | 5 | 5 | 5 | 5 |
| | P | 0.111 | 0.905 | 0.310 | 0.691 | 0.032 | 0.016 | 0.905 | 0.008 | 0.032 | 0.421 |
| 720 min | Mean ± SEM | 3.6 ± 1.1 | 34.7 ± 7.3 | 19.4 ± 5.3 | 40.4 ± 7.0 | 49.6 ± 5.3 | 2.5 ± 0.7 | 44.6 ± 11.7 | 25.8 ± 6.6 | 36.2 ± 4.9 | 37.1 ± 18.3 |
| | N | 5 | 5 | 5 | 5 | 5 | 5 | 5 | 5 | 5 | 5 |
| | P | 0.556 | 0.413 | 0.691 | 0.421 | 0.151 | 0.730 | 0.413 | 0.548 | 0.151 | 0.402 |
| 1440 min | Mean ± SEM | 4.7 ± 0.5 | 36.3 ± 9.1 | 17.5 ± 3.8 | 30.3 ± 2.2 | 38.6 ± 6.4 | 2.6 ± 0.4 | 30.3 ± 4.9 | 25.7 ± 7.6 | 32.2 ± 4.4 | 14.8 ± 2.5 |
| | N | 5 | 5 | 5 | 5 | 5 | 5 | 5 | 5 | 5 | 5 |
| | P | 0.905 | 0.111 | 0.421 | 1.000 | 1.000 | 0.623 | 0.905 | 0.841 | 0.421 | 0.548 |
| 2880 min | Mean ± SEM | 4.8 ± 0.9 | 44.3 ± 16.6 | 30.0 ± 6.0 | 40.3 ± 5.0 | 37.1 ± 4.2 | 2.2 ± 0.6 | 26.9 ± 3.5 | 23.1 ± 7.6 | 37.2 ± 5.3 | 15.4 ± 2.0 |
| | N | 6 | 6 | 6 | 6 | 6 | 6 | 6 | 6 | 6 | 6 |
| | P | 0.610 | 0.257 | 0.792 | 0.329 | 0.537 | 0.171 | 0.762 | 0.931 | 0.082 | 0.662 |
| | | | | | | | | | | | |
| Air 60 min | Mean ± SEM | 5.2 ± 1.4 | 73.9 ± 29.0 | 24.1 ± 6.6 | 31.4 ± 6.8 | 38.4 ± 1.6 | 3.4 ± 0.9 | 30.8 ± 5.0 | 20.3 ± 6.6 | 27.2 ± 3.5 | 21.9 ± 6.1 |
| | N | 4 | 4 | 5 | 5 | 5 | 4 | 4 | 5 | 5 | 5 |

## Slide 4
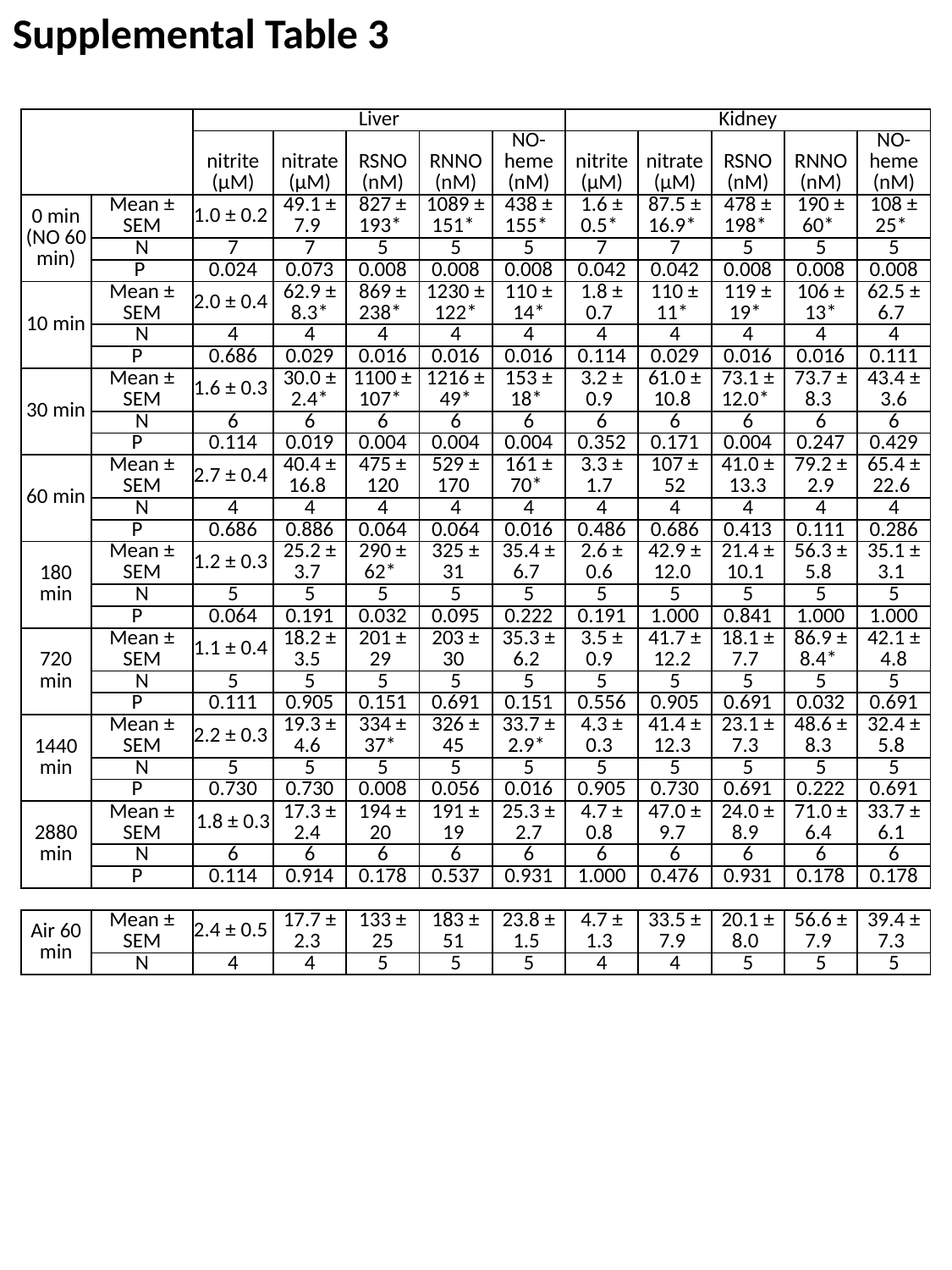

Supplemental Table 3
| | | Liver | | | | | Kidney | | | | |
| --- | --- | --- | --- | --- | --- | --- | --- | --- | --- | --- | --- |
| | | nitrite (μM) | nitrate (μM) | RSNO (nM) | RNNO (nM) | NO-heme (nM) | nitrite (μM) | nitrate (μM) | RSNO (nM) | RNNO (nM) | NO-heme (nM) |
| 0 min (NO 60 min) | Mean ± SEM | 1.0 ± 0.2 | 49.1 ± 7.9 | 827 ± 193\* | 1089 ± 151\* | 438 ± 155\* | 1.6 ± 0.5\* | 87.5 ± 16.9\* | 478 ± 198\* | 190 ± 60\* | 108 ± 25\* |
| | N | 7 | 7 | 5 | 5 | 5 | 7 | 7 | 5 | 5 | 5 |
| | P | 0.024 | 0.073 | 0.008 | 0.008 | 0.008 | 0.042 | 0.042 | 0.008 | 0.008 | 0.008 |
| 10 min | Mean ± SEM | 2.0 ± 0.4 | 62.9 ± 8.3\* | 869 ± 238\* | 1230 ± 122\* | 110 ± 14\* | 1.8 ± 0.7 | 110 ± 11\* | 119 ± 19\* | 106 ± 13\* | 62.5 ± 6.7 |
| | N | 4 | 4 | 4 | 4 | 4 | 4 | 4 | 4 | 4 | 4 |
| | P | 0.686 | 0.029 | 0.016 | 0.016 | 0.016 | 0.114 | 0.029 | 0.016 | 0.016 | 0.111 |
| 30 min | Mean ± SEM | 1.6 ± 0.3 | 30.0 ± 2.4\* | 1100 ± 107\* | 1216 ± 49\* | 153 ± 18\* | 3.2 ± 0.9 | 61.0 ± 10.8 | 73.1 ± 12.0\* | 73.7 ± 8.3 | 43.4 ± 3.6 |
| | N | 6 | 6 | 6 | 6 | 6 | 6 | 6 | 6 | 6 | 6 |
| | P | 0.114 | 0.019 | 0.004 | 0.004 | 0.004 | 0.352 | 0.171 | 0.004 | 0.247 | 0.429 |
| 60 min | Mean ± SEM | 2.7 ± 0.4 | 40.4 ± 16.8 | 475 ± 120 | 529 ± 170 | 161 ± 70\* | 3.3 ± 1.7 | 107 ± 52 | 41.0 ± 13.3 | 79.2 ± 2.9 | 65.4 ± 22.6 |
| | N | 4 | 4 | 4 | 4 | 4 | 4 | 4 | 4 | 4 | 4 |
| | P | 0.686 | 0.886 | 0.064 | 0.064 | 0.016 | 0.486 | 0.686 | 0.413 | 0.111 | 0.286 |
| 180 min | Mean ± SEM | 1.2 ± 0.3 | 25.2 ± 3.7 | 290 ± 62\* | 325 ± 31 | 35.4 ± 6.7 | 2.6 ± 0.6 | 42.9 ± 12.0 | 21.4 ± 10.1 | 56.3 ± 5.8 | 35.1 ± 3.1 |
| | N | 5 | 5 | 5 | 5 | 5 | 5 | 5 | 5 | 5 | 5 |
| | P | 0.064 | 0.191 | 0.032 | 0.095 | 0.222 | 0.191 | 1.000 | 0.841 | 1.000 | 1.000 |
| 720 min | Mean ± SEM | 1.1 ± 0.4 | 18.2 ± 3.5 | 201 ± 29 | 203 ± 30 | 35.3 ± 6.2 | 3.5 ± 0.9 | 41.7 ± 12.2 | 18.1 ± 7.7 | 86.9 ± 8.4\* | 42.1 ± 4.8 |
| | N | 5 | 5 | 5 | 5 | 5 | 5 | 5 | 5 | 5 | 5 |
| | P | 0.111 | 0.905 | 0.151 | 0.691 | 0.151 | 0.556 | 0.905 | 0.691 | 0.032 | 0.691 |
| 1440 min | Mean ± SEM | 2.2 ± 0.3 | 19.3 ± 4.6 | 334 ± 37\* | 326 ± 45 | 33.7 ± 2.9\* | 4.3 ± 0.3 | 41.4 ± 12.3 | 23.1 ± 7.3 | 48.6 ± 8.3 | 32.4 ± 5.8 |
| | N | 5 | 5 | 5 | 5 | 5 | 5 | 5 | 5 | 5 | 5 |
| | P | 0.730 | 0.730 | 0.008 | 0.056 | 0.016 | 0.905 | 0.730 | 0.691 | 0.222 | 0.691 |
| 2880 min | Mean ± SEM | 1.8 ± 0.3 | 17.3 ± 2.4 | 194 ± 20 | 191 ± 19 | 25.3 ± 2.7 | 4.7 ± 0.8 | 47.0 ± 9.7 | 24.0 ± 8.9 | 71.0 ± 6.4 | 33.7 ± 6.1 |
| | N | 6 | 6 | 6 | 6 | 6 | 6 | 6 | 6 | 6 | 6 |
| | P | 0.114 | 0.914 | 0.178 | 0.537 | 0.931 | 1.000 | 0.476 | 0.931 | 0.178 | 0.178 |
| | | | | | | | | | | | |
| Air 60 min | Mean ± SEM | 2.4 ± 0.5 | 17.7 ± 2.3 | 133 ± 25 | 183 ± 51 | 23.8 ± 1.5 | 4.7 ± 1.3 | 33.5 ± 7.9 | 20.1 ± 8.0 | 56.6 ± 7.9 | 39.4 ± 7.3 |
| | N | 4 | 4 | 5 | 5 | 5 | 4 | 4 | 5 | 5 | 5 |

## Slide 5
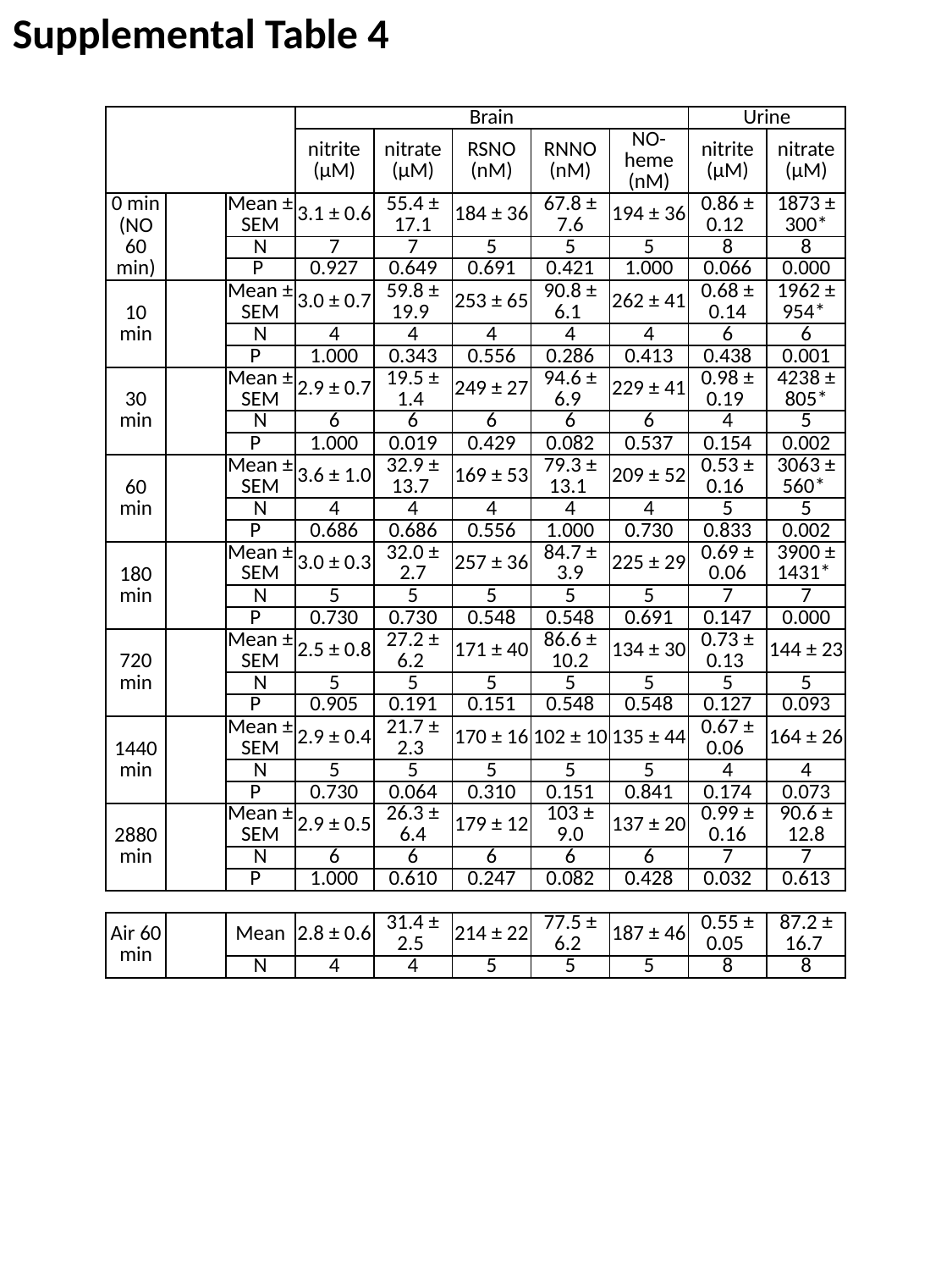

Supplemental Table 4
| | | | Brain | | | | | Urine | |
| --- | --- | --- | --- | --- | --- | --- | --- | --- | --- |
| | | | nitrite (μM) | nitrate (μM) | RSNO (nM) | RNNO (nM) | NO-heme (nM) | nitrite (μM) | nitrate (μM) |
| 0 min (NO 60 min) | | Mean ± SEM | 3.1 ± 0.6 | 55.4 ± 17.1 | 184 ± 36 | 67.8 ± 7.6 | 194 ± 36 | 0.86 ± 0.12 | 1873 ± 300\* |
| | | N | 7 | 7 | 5 | 5 | 5 | 8 | 8 |
| | | P | 0.927 | 0.649 | 0.691 | 0.421 | 1.000 | 0.066 | 0.000 |
| 10 min | | Mean ± SEM | 3.0 ± 0.7 | 59.8 ± 19.9 | 253 ± 65 | 90.8 ± 6.1 | 262 ± 41 | 0.68 ± 0.14 | 1962 ± 954\* |
| | | N | 4 | 4 | 4 | 4 | 4 | 6 | 6 |
| | | P | 1.000 | 0.343 | 0.556 | 0.286 | 0.413 | 0.438 | 0.001 |
| 30 min | | Mean ± SEM | 2.9 ± 0.7 | 19.5 ± 1.4 | 249 ± 27 | 94.6 ± 6.9 | 229 ± 41 | 0.98 ± 0.19 | 4238 ± 805\* |
| | | N | 6 | 6 | 6 | 6 | 6 | 4 | 5 |
| | | P | 1.000 | 0.019 | 0.429 | 0.082 | 0.537 | 0.154 | 0.002 |
| 60 min | | Mean ± SEM | 3.6 ± 1.0 | 32.9 ± 13.7 | 169 ± 53 | 79.3 ± 13.1 | 209 ± 52 | 0.53 ± 0.16 | 3063 ± 560\* |
| | | N | 4 | 4 | 4 | 4 | 4 | 5 | 5 |
| | | P | 0.686 | 0.686 | 0.556 | 1.000 | 0.730 | 0.833 | 0.002 |
| 180 min | | Mean ± SEM | 3.0 ± 0.3 | 32.0 ± 2.7 | 257 ± 36 | 84.7 ± 3.9 | 225 ± 29 | 0.69 ± 0.06 | 3900 ± 1431\* |
| | | N | 5 | 5 | 5 | 5 | 5 | 7 | 7 |
| | | P | 0.730 | 0.730 | 0.548 | 0.548 | 0.691 | 0.147 | 0.000 |
| 720 min | | Mean ± SEM | 2.5 ± 0.8 | 27.2 ± 6.2 | 171 ± 40 | 86.6 ± 10.2 | 134 ± 30 | 0.73 ± 0.13 | 144 ± 23 |
| | | N | 5 | 5 | 5 | 5 | 5 | 5 | 5 |
| | | P | 0.905 | 0.191 | 0.151 | 0.548 | 0.548 | 0.127 | 0.093 |
| 1440 min | | Mean ± SEM | 2.9 ± 0.4 | 21.7 ± 2.3 | 170 ± 16 | 102 ± 10 | 135 ± 44 | 0.67 ± 0.06 | 164 ± 26 |
| | | N | 5 | 5 | 5 | 5 | 5 | 4 | 4 |
| | | P | 0.730 | 0.064 | 0.310 | 0.151 | 0.841 | 0.174 | 0.073 |
| 2880 min | | Mean ± SEM | 2.9 ± 0.5 | 26.3 ± 6.4 | 179 ± 12 | 103 ± 9.0 | 137 ± 20 | 0.99 ± 0.16 | 90.6 ± 12.8 |
| | | N | 6 | 6 | 6 | 6 | 6 | 7 | 7 |
| | | P | 1.000 | 0.610 | 0.247 | 0.082 | 0.428 | 0.032 | 0.613 |
| | | | | | | | | | |
| Air 60 min | | Mean | 2.8 ± 0.6 | 31.4 ± 2.5 | 214 ± 22 | 77.5 ± 6.2 | 187 ± 46 | 0.55 ± 0.05 | 87.2 ± 16.7 |
| | | N | 4 | 4 | 5 | 5 | 5 | 8 | 8 |

## Slide 6
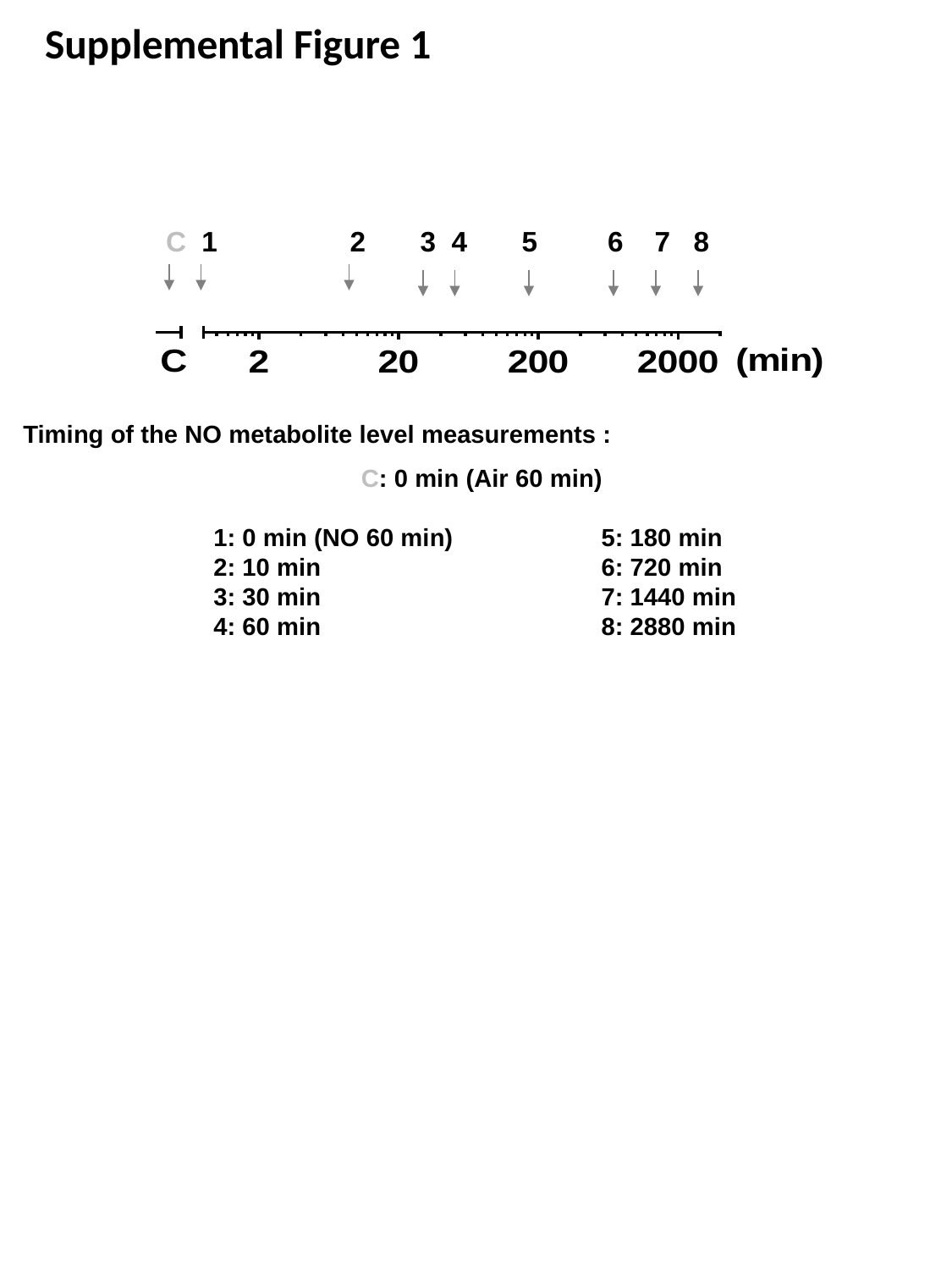

Supplemental Figure 1
C 1 2 3 4 5 6 7 8
Timing of the NO metabolite level measurements :
	 C: 0 min (Air 60 min)
1: 0 min (NO 60 min)		 5: 180 min
2: 10 min 			 6: 720 min
3: 30 min 			 7: 1440 min
4: 60 min 			 8: 2880 min

## Slide 7
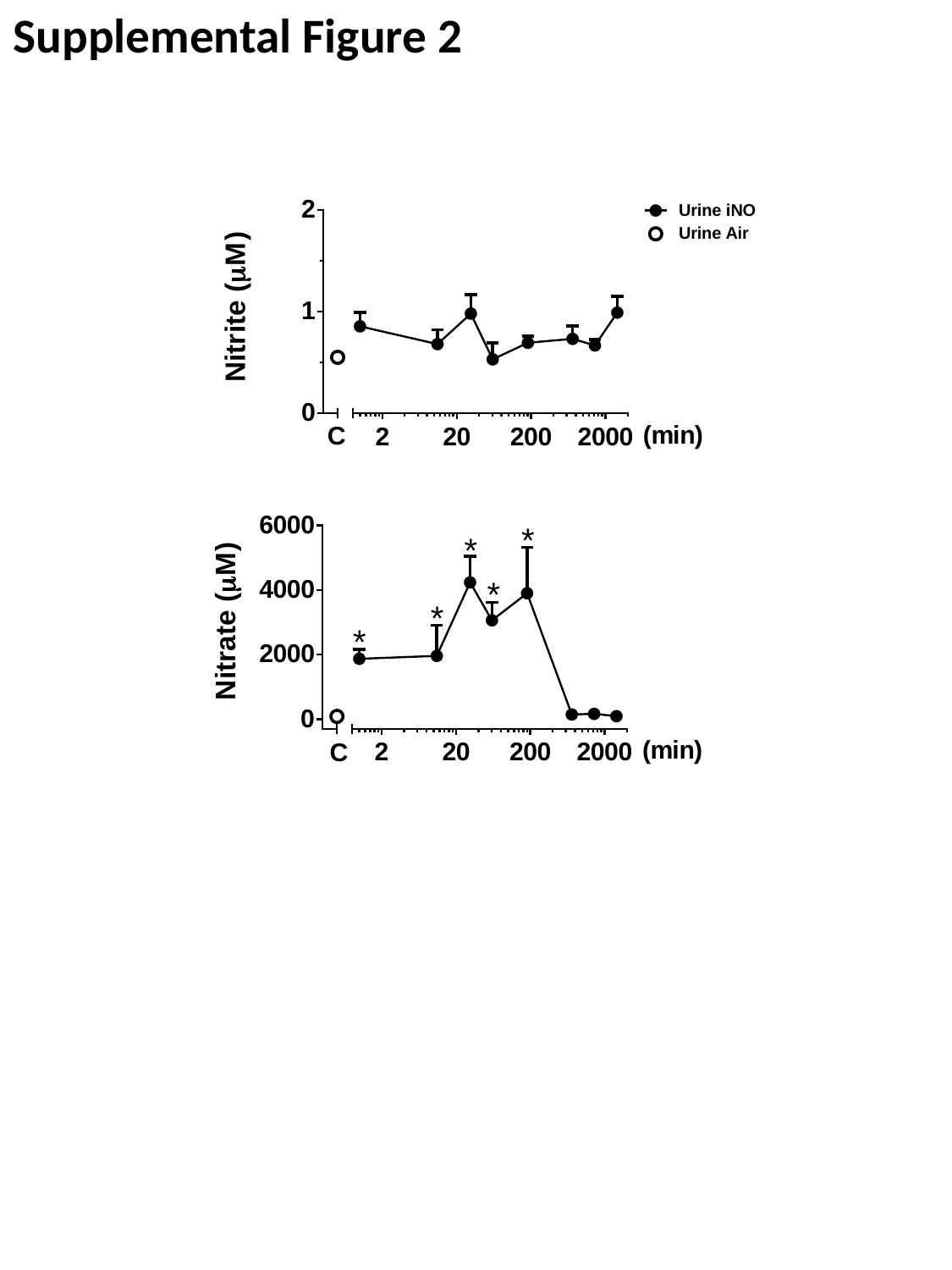

Supplemental Figure 2

## Slide 8
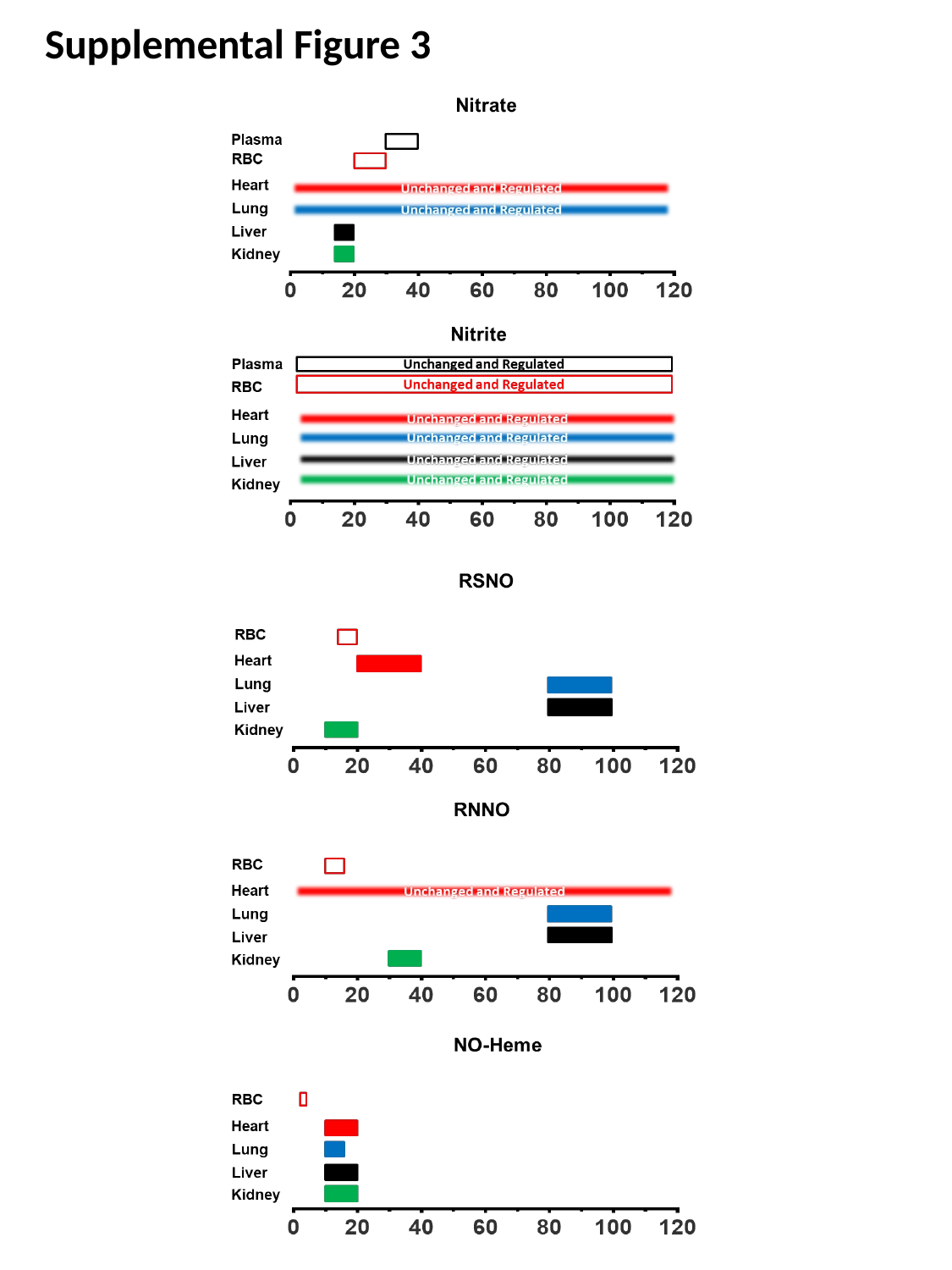

Supplemental Figure 3
